# Copper homeostasis and cuproptosis in health and disease

**DOI:** 10.1038/s41392-022-01229-y

**Published:** 2022-11-23

**Authors:** Liyun Chen, Junxia Min, Fudi Wang

**Affiliations:** 1 The Fourth Affiliated Hospital, The First Affiliated Hospital, Institute of Translational Medicine, School of Public Health, State Key Laboratory of Experimental Hematology, Zhejiang University School of Medicine, Hangzhou, China; 2 The First Affiliated Hospital, Basic Medical Sciences, School of Public Health, Hengyang Medical School, University of South China, Hengyang, China

**Keywords:** Physiology, Molecular medicine

## Abstract

As an essential micronutrient, copper is required for a wide range of physiological processes in virtually all cell types. Because the accumulation of intracellular copper can induce oxidative stress and perturbing cellular function, copper homeostasis is tightly regulated. Recent studies identified a novel copper-dependent form of cell death called cuproptosis, which is distinct from all other known pathways underlying cell death. Cuproptosis occurs via copper binding to lipoylated enzymes in the tricarboxylic acid (TCA) cycle, which leads to subsequent protein aggregation, proteotoxic stress, and ultimately cell death. Here, we summarize our current knowledge regarding copper metabolism, copper-related disease, the characteristics of cuproptosis, and the mechanisms that regulate cuproptosis. In addition, we discuss the implications of cuproptosis in the pathogenesis of various disease conditions, including Wilson’s disease, neurodegenerative diseases, and cancer, and we discuss the therapeutic potential of targeting cuproptosis.

## Introduction

The essential micronutrient copper (Cu) functions as a key catalytic cofactor in a wide array of biological processes, including mitochondrial respiration, antioxidant defense, and bio-compound synthesis. Importantly, the intracellular Cu concentration is kept at a relatively low range, and moderate increases can cause cytotoxicity and even lead to cell death; thus, the uptake, distribution, and elimination of Cu are tightly regulated. Moreover, in humans, genetic mutations that cause Cu accumulation have been linked to severe, potentially life-threatening pathological conditions.^1^ Thus, understanding the process by which Cu accumulation causes cellular toxicity is an important first step toward developing new, effective therapies.

Cuproptosis is a recently discovered form of regulated cell death triggered by excess Cu^2+^.^2^ This Cu-induced form of programmed cell death is distinct from other cell death pathways, including apoptosis, ferroptosis, and necroptosis. Intracellular Cu targets and binds to lipoylated components in the tricarboxylic acid (TCA) cycle, and aggregation of these Cu-bound lipoylated mitochondrial proteins—and the subsequent reduction in Fe–S (iron–sulfur) clusters—induces proteotoxic stress and ultimately cell death.

Here, we review our current knowledge regarding the features of cuproptosis and the underlying mechanisms. In addition, we discuss new perspectives with respect to the putative pathophysiological role of cuproptosis in various diseases, as well as the therapeutic potential of targeting this novel form of cell death.

## Systemic copper metabolism

Cu is an essential element in virtually all living organisms, and numerous studies have shown that Cu serves as a cofactor for a variety of key metabolic enzymes that drive a broad range of physiological processes.^3,4^ Thus, the systemic Cu levels must be maintained within a narrow range to ensure normal biochemical processes.

The absorption of dietary Cu occurs primarily at the duodenum and small intestine.^5^ The uptake of Cu into intestinal epithelial cells is mediated mainly by Cu transport protein 1 (CTR1), located on the apical side of the enterocytes. This process is facilitated by the activity of the metalloreductases six-transmembrane epithelial antigen of the prostate (STEAP) and duodenal cytochrome b (DCYTB),^6,7^ which reduce divalent Cu^2+^ to monovalent Cu^+^; the ionic state in which CTR1 transports Cu. Following absorption by the gastrointestinal tract, Cu is secreted into the bloodstream and bound to soluble chaperones, such as albumin, transcuprein, histidines, and macroglobulins.^8–11^ Upon reaching the liver, the hepatocytes mediate the uptake of Cu via CTR1. Inside the cytoplasm, Cu is then either delivered by Cu chaperones to specific proteins or chelated by metallothionein (MT) for storage.^12–15^ The principal Cu chaperones include COX17 (which delivers Cu to cytochrome c oxygenase), CCS (Cu chaperone for superoxide dismutase, which delivers Cu to superoxide dismutase 1), and ATOX1 (which delivers Cu to ATP7A and ATP7B). The Cu-ATPases (ATP7B in the case of hepatocytes) pump Cu ions from the liver back into the blood, where it again binds to soluble chaperones and is transported to specific tissues and organs.^16^ Upon reaching its target tissues, Cu catalyzes reactions in a wide range of physiological processes, including mitochondrial energy production, tyrosine and neurotransmitter metabolism, redox homeostasis, and extracellular matrix remodeling.^17–20^

In the body, Cu is stored primarily in the liver,^21,22^ and excess of Cu is eliminated in the feces, either through biliary excretion—the major form of endogenous Cu elimination—or as unabsorbed metal ions.^23^ Other pathways for Cu elimination such as urine, sweat, and menses play a relatively minor role in Cu loss. Taken together, the systemic Cu status is regulated by duodenal absorption and/or biliary excretion. In the context of high Cu intake, the absorption of Cu decreases and elimination of Cu increases; conversely, during periods of low Cu intake, endogenous Cu excretion via the bile decreases, and the retention of absorbed Cu increases.^24^

## Copper homeostasis is tightly regulated in cells

To maintain Cu homeostasis at the cellular level, intracellular Cu content is regulated by a complex network of Cu-dependent proteins, including cuproenzymes, Cu chaperones, and membrane transporters. These proteins work together to coordinate the import, export, and intracellular utilization of Cu, thus maintaining cellular Cu levels within a specific range, which helps prevent the consequences of Cu overload and Cu deficiency.

### Cu uptake into cells

**Fig. 1:**
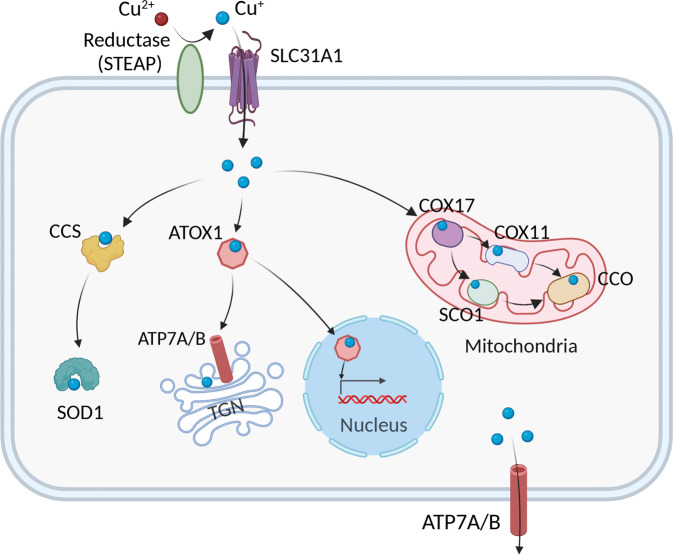
Summary of the pathways that mediate cellular Cu metabolism. Extracellular Cu^2+^ is reduced by the reductase STEAP to Cu^+^, which is transported into the cell by the Cu transporter CTR1, where it is delivered to cytosolic Cu chaperones such as CCS and SOD1 and then delivered to specific subcellular compartments such as the mitochondria, TGN, and nucleus. In the mitochondria, Cu is involved in the respiratory chain and redox pathways via binding to CCO. In the mitochondrial intermembrane space, COX17 binds to and delivers Cu to either SCO1 or COX11, which transfers Cu to the cytochrome oxidase subunit. In the nucleus, Cu can bind to transcription factors and drive gene expression. Finally, in the TGN the Cu^+^-ATPase transporters ATP7A and ATP7B transfer Cu from the cytosol to the TGN lumen, where it activates Cu-dependent enzymes in the secretory pathway. When cytosolic Cu levels are high, ATP7A and ATP7B exit the TGN and facilitate Cu export. Created with BioRender. ATOX1, antioxidant 1 copper chaperone; ATP7A and ATP7B, ATPase copper transporter 7A and 7B, respectively; CCO, cytochrome c oxidase; CCS, copper chaperone for superoxide dismutase; COX17 cytochrome c oxidase copper chaperone 17, COX11 cytochrome c oxidase copper chaperone 11, SCO1 synthesis of cytochrome c oxidase 1, SOD1 superoxide dismutase 1, STEAP the six-transmembrane epithelial antigen of the prostate, SLC31A1 solute carrier family 31 member 1, TGN trans-Golgi network

The high-affinity Cu transporter CTR1 (also known as SLC31A1) is structurally and functionally conserved from yeast to humans and is responsible for the majority of Cu uptake into cells.^25^ A growing body of evidence suggests that CTR1 transports Cu^+^, and Ctr1 may function together with metalloreductases such as STEAP and/or DCYTB to transport Cu across the membrane^26,27^ (Fig. 1). Moreover, in vitro studies have shown that the expression of CTR1 is regulated in a Cu-dependent manner, in which it is down-regulated under Cu-overload conditions and up-regulated under Cu-depleted conditions.^28^ Similarly, increased expression of Ctr1 has been observed in the intestines of mice fed a Cu-deficient diet, suggesting a negative-feedback loop of Ctr1 regulation.^29^

Several lines of evidence suggest that CTR1 is required for the transport of Cu into specific organs/tissues. For example, intestine-specific Ctr1 knockout mice develop Cu deficiency in the majority of peripheral tissues, suggesting that Ctr1 functions as the major factor driving intestinal Cu absorption.^30^ Interestingly, global Ctr1 global knockout (Ctr1^–/–^) mice die in utero a mid-way through gestation, and embryonic fibroblasts prepared from Ctr1^–/–^ embryos have drastically reduced levels of Cu and Cu-dependent enzyme activity, suggesting that Ctr1 plays an essential role in embryonic development in mammals.^31^

### Intracellular Cu distribution

Within the cytoplasm, Cu trafficking is tightly coordinated by a fine-tuned network of high-affinity Cu chaperones. The chaperone antioxidant-1 (ATOX1) is responsible for transferring Cu to ATP7A and ATP7B in the trans-Golgi network (TGN) and facilitates the synthesis of cuproenzymes such as lysyl oxidase, tyrosinase, and ceruloplasmin^32^ (Fig. 1). In embryonic mouse fibroblasts, Atox1 has also been shown to serve as a Cu-dependent transcriptional regulator, and contribute to cell proliferation.^33^ Mice lacking the gene encoding Atox1 are observed to be perinatal lethal due to impaired Cu balance, which likely reflects the central role that this chaperone plays in Cu transport and homeostasis.^34^

The chaperone CCS delivers Cu to superoxide dismutase 1 (SOD1) to detoxify ROS and maintain Cu homeostasis (Fig. 1). Studies have shown that organisms lacking SOD1 have increased oxidative stress. For example, yeast with mutated *sod1* accumulate DNA mutations,^35^ and SOD1 knockout mice develop hepatocellular carcinoma, presumably as a result of oxidative damage of hepatocytes.^36^ Moreover, CCS expression is regulated by cellular Cu status; when Cu content decreases, CCS levels increase, and when Cu content is high, the degradation of CCS increases.^37,38^ Both CCS and SOD1 are localized in both the cytoplasm and mitochondrial intermembrane space (IMS), where they detoxify mitochondria-derived superoxides.^39,40^

In addition to delivering Cu to the secretory compartment and cytosolic proteins, Cu is targeted to the mitochondria, where cytochrome oxidase (COX) uses Cu for oxidative phosphorylation and mitochondrial function. In humans, COX consists of two core subunits, COX1 and COX2, which bind Cu at the conserved Cu_B_ and Cu_A_ sites, respectively.^41^ The Cu chaperone COX17, located in the IMS, is responsible for transporting Cu from the cytosol to the mitochondrial IMS and contributes to the assembly of COX.^15,42^ In the IMS, Cu^+^ is bound to COX17 and delivered either to the chaperone synthesis of cytochrome c oxidase 1 (SCO1) for transfer to the COX2 subunit, or to COX11 for delivery to the COX1 subunit^43–45^ (Fig. 1). Finally, mutations in COX17, SCO1, and SCO2 have been associated with decreased COX activity and can even be fatal.^46,47^

### Cu efflux mediated by Cu-ATPases

The Cu-ATPases ATP7A and ATP7B act as the major transporter for exporting cellular Cu. The regulation of ATP7A/7B localization and function is important for mediating this process and is multifaceted^48^ (Fig. 1). Under physiological cellular Cu levels, these transporters are found to be located in the TGN where they pump Cu from the cytosol into the lumen of the TGN. When intracellular Cu increases, these transporters translocate from the TGN to vesicular compartments and fuse with the plasma membrane to export Cu; when Cu content returns to physiological levels, they are recycled back to the TGN.^49^

ATP7A and ATP7B have distinct expression patterns. ATP7A is expressed in most tissues/organs, with the exception of the liver, where ATP7B is found to be predominantly expressed.^32^ At the basolateral membrane of enterocytes, Cu is pumped by ATP7A into the portal circulation for delivery to the liver, the main organ for Cu storage.^50^ In other tissues such as the placenta and the blood–brain barrier (BBB), ATP7A mediates the transport of Cu across polarized cells to ensure adequate Cu is delivered for the development of the fetus and brain.^32^ In the liver, ATP7B mobilizes Cu in secretory vesicles to the bile in order to prevent the accumulation of Cu. Consistent with their important roles in maintaining health, mutations in ATP7A and ATP7B cause inherited disorders of Cu metabolism, which have been recognized as Menkes disease (MD) and Wilson’s disease (WD), respectively.^51^

## Diseases related to copper dysregulation

Given that either Cu deficiency or accumulation exhibit adverse health effect, it is important to note that the inability to maintain Cu balance has been associated with a wide range of pathologic conditions, including MD, WD, neurodegenerative diseases, cancer, and cardiovascular disease.

### Menkes disease

MD is an X-linked recessive disease that affects Cu metabolism and is caused by mutations in the *ATP7A* gene.^52^ The resulting loss of functional ATP7A in the intestine leads to reduced Cu efflux into the blood, an accumulation of Cu in enterocytes, and generalized severe systemic Cu deficiency.^53^ MD is a fatal disorder, and affected patients usually die in early childhood. Children with MD exhibit severe symptoms such as mental retardation, hypothermia, neuronal degeneration, bone fractures, hair and skin abnormalities, and widespread vascular abnormalities.^51^

In the brain of patients with MD, the deficiency of Cu adversely affects the physiological function of Cu-dependent enzymes such as dopamine-β-hydroxylase (DβH), which is essential for synthesizing the neurotransmitter.^54^ Infants with MD present with high levels of dihydroxyphenylalanine, the precursor of catecholamines, suggesting reduced DβH activity.^54,55^ The impaired function of DβH may lead to impaired synaptogenesis and axonal growth, manifesting as hypotonia and seizures. In addition, impaired mitochondrial function and intracerebral lactic acidosis caused by reduced COX activity in the brain may also contribute to the degeneration of neurons in these patients.^56^

Low levels of Cu have also been reported in the heart, liver, muscle, skin, and connective tissues of patients with MD. For example, Cu deficiency in the heart has correlated with congenital cardiovascular abnormalities such as tetralogy of Fallot, which can present in newborn infants with MD.^57^ In the skin, muscle, and connective tissues, reduced ATP7A function results in decreased activity of lipoxygenase (LOX), which oxidizes the lysine and hydroxylysine in elastin and collagen.^58^ Decreased LOX activity reduces the formation of the covalent cross-links that provide tensile strength and elasticity to skeletal, muscular, and cardiovascular connective tissues.^59^ Thus, patients with MD often present with abnormalities in their connective tissue, including osteopenia, skin laxity, arterial aneurysms, and spontaneous fractures.^60^

Currently, patients with MD are diagnosed primarily through a genetic screen and treated using Cu salts such as Cu-histidine complexes.^61,62^ Subcutaneously injected Cu-histidinate delivers Cu directly through the bloodstream to various tissue/organs, bypassing the malfunctioning mechanism by which Cu is normally absorbed through the intestine, thereby helping restore systemic Cu levels in patients with MD.^61,63^ Notably, treatment with Cu-histidine has been shown to be effective in patients in which the mutated ATP7A protein retains the ability to transport Cu across the BBB.^64^ In addition, patients who are treated with Cu-histidine in an early stage of the disease can exhibit gross improvements, with fine neuronal development and motor movement, whereas patients who are diagnosed and treated in a later stage generally have a poor prognosis.^64^ Moreover, treatment with Cu-histidine appears to result in a better general neurological outcome in asymptomatic patients compared to symptomatic patients.^65^

### Wilson disease

A classic example of a Cu-overload condition is WD, an autosomal recessive disorder characterized by a variety of mutations in the *ATP7B* gene. The resulting impaired ATP7B function impairs Cu excretion and leads to persistent Cu accumulation in the brain, liver, and other tissues.^66^ In healthy individuals, hepatic Cu content is generally <55 μg/g dry weight; in patients with WD, hepatic Cu can exceed 250 μg/g dry weight.^67^ Most frequently, WD patients present with hepatic and/or neuropsychiatric symptoms.

Cu toxicity is considered as the primary cause of organ damage in patients with WD.^68–70^ Moreover, DNA damage, lipid peroxidation, and dysfunction of mitochondrial are typical findings in the liver of patients with WD.^71,72^ Morphological changes in hepatic mitochondria—including widening of the interstitial space, separation and enlargement of the inner and outer membranes, and/or the occurrence of large vacuoles—usually occur in the early stages of WD, and these changes are considered a hallmark feature of hepatic injury in WD.^67^ Patients with WD can present with liver symptoms that include fulminant hepatic failure (also known as acute liver failure), persistently elevated levels of serum aminotransferases, jaundice, and chronic hepatitis.^73^ Interestingly, some patients do not manifest these hepatic changes, but instead present with neurological symptoms; however, hepatic Cu accumulation has also been observed in these particular patients and may lead to the development of hepatic cirrhosis.^74^ In most cases of WD, Cu deposition can be observed in the cornea known as Kayser–Fleischer rings, representing as an ophthalmic manifestation of the disease.^66^

Approximately 40–50% of patients with WD present with neurological and neuropsychiatric signs.^75^ Moreover, high levels of Cu have been observed in nearly all brain regions in patients with WD.^76^ The main neurological symptoms include tremors, akinetic-rigid syndrome (Parkinsonism), ataxia, and dystonia; other common neurological findings can include dysarthria, spasticity, and a lack of motor coordination.^77^ Structural brain MRI scans in patients with MD have shown widespread lesions in the midbrain, putamen, pons, globus pallidus, thalamus, and cerebellum, as well as cortical atrophy.^67,78^ Loss of neurons and the occurrence of abnormal astrocytes are typical neuropathological features in patients with WD.^79^ Non-hepatic and non-neuronal complications associated with WD include osteomalacia, osteoarthritis, hemolysis, cardiac arrhythmia, and renal abnormalities.^80–82^

Evidence is limited regarding the strategy of restricting dietary Cu for treating WD.^83^ Current strategies for treating WD primarily focus on oral zinc to decrease Cu absorption and the use of chelating agents such as d-penicillamine and trientine.^84–86^ The choice of treatment generally depends on the disease stage, with Cu chelators recommended for patients with advanced symptomatology.^84,87^ Weiss et al. previously evaluated the efficacy of the chelators d-penicillamine and trientine in patients with WD; they found that although treatment led to hepatic improvement in >90% of patients, the response rate among neurological patients was considerably less favorable.^88^ However, after 4 years of therapy, around 60% of patients showed improvement in neurological symptoms.^88^ Another chelating agent, tetrathiomolybdate (TTM), has also been shown to reduce circulating levels of free Cu ions.^89^ A randomized trial comparing the efficacy of TTM versus trientine in patients with neurological WD has found that neurological deterioration was less common in the TTM-treated group.^86^ In addition, another clinical trial has shown that the use of WTX101, the bis-choline salt of TTM, exhibits beneficial effects and significant improvements in controlling Cu-induced neurological symptoms.^90^

### Neurodegenerative diseases

#### Alzheimer’s disease (AD)

AD is one of the most common neurodegenerative disorders. The pathological hallmark of AD is the deposition of amyloid plaques and neurofibrillary tangles in the gray matter due to aberrant processing of amyloid precursor protein (APP) leading to the aggregation of amyloid-β (Aβ) peptides and tau protein, respectively.^91^

Mounting evidence has implicated that altered Cu homeostasis may be associated with the pathogenesis of AD, as Cu may interact with several key pathogenic factors such as Aβ and tau. Moreover, serum-free Cu levels are known to be elevated with aging,^92^ and both total and free Cu levels are increased in the serum of patients with AD compared to healthy controls.^93–95^ Importantly, high levels of Cu have been measured in senile plaques in patients with AD.^96^ Excess Cu could bind directly to Aβ peptides with high affinity, further increasing aggregation of Aβ and driving increased neurotoxicity.^97,98^ Binding of Cu to Aβ leads to the formation of neurotoxic dityrosine-linked β-amyloid dimers, which prevents the dimers from degrading to monomers and may therefore be relevant to the formation of amyloid deposits.^97,99,100^ In vitro, sequestration of Cu from Aβ peptides prevents its accumulation and leads to degradation of Aβ, suppression of hydroxyl radical (•OH) production, and oxidative damage, eventually reducing cell death.^101–103^

Cu also plays an essential role in the activation of microglia, a process linked to neurodegeneration. In the BV2 cells (a mouse microglial cell line), Cu could trigger activation of the NF-κB signaling pathway and increase the release of inflammatory factors such as nitric oxide (NO) and the cytokine tumor necrosis factor-α (TNF-α).^104^ Cu in excess may also reduce the brain’s physiological ability to remove Aβ peptides.^105^ Kitazawa et al. have reported that the existence of Cu-Aβ complexes reduce the expression of LRP1 (lipoprotein receptor-related protein 1), further reducing the clearance of neurotoxic Aβ and increasing brain Aβ deposits.^104^ Moreover, Cu may play a pathogenic role in tau proteins in the context of AD. For example, Cu could trigger the phosphorylation and aggregation of tau protein, enhancing the neurotoxicity of tau aggregates.^106,107^

Aiming to evaluate the effect of lowering-Cu therapy in treating AD, several Cu chelators have been developed and tested in mouse models of AD, showing promising beneficial effects such as antioxidant properties, reduced Aβ aggregation, and amelioration of neurological symptoms. For example, using an APP transgenic mouse model of AD, Cherny et al. have shown that the chelator clioquinol could decrease Aβ deposits and improve the capacities in both learning and memory.^101^ Moreover, several phase II trials have shown that clioquinol is capable of reducing Aβ aggregation and improving cognitive function, but fail to provide sufficient evidence of a positive benefit in a larger trial.^108,109^ The clioquinol derivative PBT2 has been shown to inhibit Cu-induced Aβ accumulation and possess greater BBB permeability and solubility.^110,111^ In a mouse model of AD, the treatment of PBT2 decreases the level of interstitial brain Aβ and the phosphorylation level of tau protein, and lead to the restoration of cognition.^108^ Finally, positive results have also been obtained from several phase Ib/IIa trials, which demonstrate that PBT2 could reduce Aβ levels and improve cognitive performance in patients with AD.^112,113^

#### Amyotrophic lateral sclerosis

Amyotrophic lateral sclerosis (ALS) is a progressive disease characterized by the selective degeneration of motor neurons leading to muscular weakness, muscle atrophy, and eventually death. Approximately 10–20% of cases present as an autosomal dominant familial form of ALS (FALS), with the remaining cases presenting as a non-inherited, sporadic form of ALS (SALS). One of the principal causes of FALS is the mutation in the *SOD1* gene.^114^ In the context of ALS, the interaction of CCS with mutant SOD1 is faulty, resulting in decreased delivery of Cu to the mitochondria and the accumulation of the less-stabilized SOD1, which is prone to acquire pro-oxidants, consequently causing a toxic gain-of-function effect in motor neurons.^115^ Interestingly, a previous study has shown that overexpressing CCS accelerates neurological deficits and shortens the life span of mice carrying the G93A mutation in SOD1 (*SOD1*^*G93A*^ mice).^116^

The role of Cu in the pathogenesis of ALS is unclear and currently under study. For example, Cu deficiency has been shown to promote the aberrant hydrophobicity of mutant SOD1, contributing to the impaired interaction of SOD1 and its neuronal toxicity, and this effect could be reversed by the supplementation of Cu.^117,118^ In the spinal cord of *SOD1*^*G37R*^ mutant mice, more than 50% of the mutant SOD1 protein is not fully Cu-bound, and treatment with a Cu-containing compound significantly decreased the levels of metal-deficient SOD1 and increased the pool of fully Cu-bound SOD1.^119^ Additional studies have shown that aggregated mutant SOD1 has low Cu content both in cultured cells and in the spinal cord of transgenic SOD1 mice, regardless of the mutant protein’s ability to bind Cu^120,121^; moreover, the degree of Cu deficiency in mutant SOD1 aggregates is proportional to the clinical severity of ALS.^122^

In addition, the dysregulated cellular Cu homeostasis and impaired function of Cu-dependent enzymes may also contribute to the toxic effects of mutant SOD1 proteins and disease progression. For example, in *SOD1*^*G93A*^-mutant mice, elevated concentrations of Cu have been observed at the presymptomatic stage in skeletal muscle and the spinal cord, and this condition aggravates further during disease progression.^123,124^ Elevated Cu levels have also been observed in the cerebrospinal fluid of patients with ALS.^121,125^ Further, as discussed above overexpressing CCS in *SOD1*^*G93A*^ mice accelerate disease progression, and this effect could be attenuated by treating the mice with a complex that delivers Cu.^116,126^ Specifically, CCS-overexpressing *SOD*^*G93A*^ mice exhibit significantly decreased COX activity when these mice reached the end stage of disease, whereas *SOD*^*G93A*^ mice not overexpressing CCS had no apparent decrease in COX activity at the same stage,^126^ supporting the hypothesis that overexpressing CCS increases the delivery of Cu to SOD1 while reducing the delivery of Cu to other enzymes (e.g., mitochondrial cytochrome c oxidase), thus leading to additional toxic effects.

It is interesting to note both Cu chelators such as d-penicillamine and TTM and Cu-delivery agents such as Cu^II^(atsm) have shown beneficial effects in several mouse models.^119,127,128^ As an example, treatment with Cu^II^(atsm) has been shown to improve motor function and increase survival in both the *SOD1*^*G93A*^ and *SOD1*^*G37R*^ mouse models of ALS.^119,129,130^ In *SOD1*^*G37R*^ mice, Cu^II^(atsm) increased the delivery of Cu to the metal-deficient mutant form of apo-SOD1, allowing its conversion to the more stable, less toxic holo-SOD1 form.^119^ Treatment with TTM could prolong the survival of both presymptomatic and symptomatic *SOD1*^*G93A*^ mice, decrease the loss of motor neurons and attenuate the severity of skeletal muscle atrophy. Notably, TTM is capable of both suppressing the activity and reducing the aggregation of mutant SOD1 proteins.^119,131^ Chelating Cu using d-penicillamine has also been reported to have beneficial effects in transgenic *SOD1* mouse models, delaying disease progression and prolonging survival.^127^

#### Huntington’s disease (HD)

HD is a rare autosomal-dominant neurological disorder characterized by a progressive loss of psychiatric, cognitive, and motor function. HD has been hypothesized to be caused by an abnormal expansion of the polyglutamine repeat at the N-terminus of the mutant Huntingtin (HTT) protein, leading to brain atrophy primarily within the striatum and cerebral cortex.^132^ The formation of aggregated mutant HTT proteins in neurons leads to reduced energy production, oxidative stress, and neurodegeneration.^133,134^

Cu has been suggested to play a role in HD. Abnormally high concentrations of Cu have been found in the striatum in patients with HD and in mouse models of HD.^135,136^ Moreover, several reports have suggested that an accumulation of Cu promotes aggregation of the HTT protein and interacts with histidine residues at the N-terminus of the protein.^136^ In vitro studies have shown that Cu binds to HTT proteins with 17–68 glutamine residues, whereas neither Fe nor Zn exhibits binding affinity. In addition, Cu chelators can inhibit the formation of mutant HTT aggregates, whereas Cu supplementation promotes aggregate formation.^136^ Moreover, Cu may contribute to the progression of HD by inhibiting mitochondrial dehydrogenases, including succinate dehydrogenase (SDH) and lactate dehydrogenase (LDH), both of which are susceptible to Cu-mediated inactivation.^137,138^ In the brain, LDH is critical for lactate metabolism,^139^ and neurons use lactate released by astrocytes as an energy substrate. In the striatum of patients with HD, reduced lactate clearance has been measured using magnetic resonance spectroscopy,^140^ reminiscent of the neuronal degeneration observed in mice treated with LDH inhibitors.^136^ These findings suggest that Cu may contribute to the pathogenesis of HD by inhibiting key enzymes involved in lactate metabolism.

With respect to treatment options, the use of Cu chelators, Clioquinol, or tetrathiomolybdate, mitigates the pathology and behavioral abnormalities of the R6/2 HD mouse model.^141,142^ In addition, the dietary intervention of Cu uptake with the treatment of bathocuproine disulfonate (BCS), the water-soluble Cu chelator, could significantly improve the survival rate in a *Drosophila* model of HD.^143^

### Cancer

#### Cu status in cancer

The role of Cu in the progression of cancer has long been a topic of study, as Cu ions may be involved in the activation of cell proliferation-related signaling pathways. Evidence has suggested that cancer cells generally have a higher demand for Cu compared to healthy, resting cells.^144^ Moreover, increased Cu concentrations have been reported in tumor tissues and/or serum obtained from patients with various types of cancers, including breast,^145,146^ lungs, gastrointestinal,^147^ oral,^148^ thyroids,^149^ gall bladder,^150^ gynecologic,^151,152^ pancreatic,^153^ and prostate cancer.^154^ In addition, elevated levels of serum Cu have been correlated with both tumor stage and disease progression in patients with colorectal, lung, and breast cancer.^155–157^

#### The role of Cu in carcinogenesis

Given that copper acts as a key factor in cellular signaling, it is not surprising that Cu is involved in the development and progression of cancer by promoting cell proliferation, angiogenesis, and metastasis.

A plethora of studies investigated the putative effects of Cu on the growth of cancer cells. For instance, daily administration of Cu sulfate (CuSO_4_) has been shown to increase tumor growth in a rat model of chemically induced mammary tumorigenesis.^158^ In a mouse model of pancreatic islet cell carcinoma, chronic exposure to elevated levels of Cu by adding 20 μM CuSO_4_ to the drinking water also accelerated tumor growth.^159^ Mice bearing BRAF^V600E^-driven lung cancer also have increased tumor proliferation when supplemented with high levels of Cu.^160^ Mechanistically, Cu can increase the production of ROS, which are intrinsically linked to malignant cell transformation. Moreover, studies have shown that CTR1-dependent Cu import activates the mitogen-activated protein kinase (MAPK) signaling cascade.^161^ In addition, Cu directly binds to MEK1 with high affinity, which in turn promotes tumor growth by activating downstream ERK1/2 phosphorylation.^160,162^ Notably, Cu can drive carcinogenesis via its functional role as an essential regulator of the autophagic kinases ULK1/2. The loss of Ctr1 results in impaired ULK1 activation and its downstream signaling, thus reducing the growth and survival of xenografted *KRAS*^*G12D*^ lung tumors.^163^

Angiogenesis, a process that involves the migration and proliferation of endothelial cells, as well as the formation of the vascular tube and new blood vessels, acts as an important factor in tumor progression. The notion that Cu possesses pro-angiogenic properties was first raised by McAuslan, who found that Cu salts could induce endothelial cell migration, an early step in angiogenesis.^164^ In support of this hypothesis, delivering Cu to the cornea in rabbits could induce the formation of new blood vessels, and Cu has been shown to enhance the proliferation and mobility of endothelial cells.^165^ In in vitro studies, silencing *CTR1* expression in endothelial cells using siRNA blocks Cu entry, decreases cell migration and reduces vascular tube formation.^166^ The pro-angiogenic properties of Cu may be attributed to its ability to regulate various factors involved in angiogenesis. For instance, Cu could regulate the secretion of angiogenic molecules such as fibroblast growth factor (FGF) and the inflammatory cytokine IL-1α.^167,168^ Cu can also modulate the affinity of angiogenin with endothelial cells by binding directly to this angiogenic factor.^169^ Deficiency of Cu has been shown to suppress transcriptional activity of NF-κB, thus inhibiting the expression of pro-angiogenic factors such as bFGF, vascular endothelial growth factor (VEGF), IL-8, IL-6, and IL-1α.^170^ Cu is also required for the activation of hypoxia-inducible factor 1 (HIF-1) activity, and the chelation of Cu blocks the HIF-1-mediated expression of VEGF, a potent angiogenic factor.^171,172^ Overexpressing the Cu-binding protein SOD1 markedly increases the production of VEGF and enhances angiogenesis induced by FGF and tumor development. Similarly, the Cu chaperone ATOX1 has also been found to serve as a modulator of angiogenesis, the depletion of Atox1 inhibit the migration of vascular smooth muscle cell that is stimulated by platelet-derived growth factor (PDGF), suggesting that ATOX1 likely plays a role in vascular remodeling and tumor angiogenesis.^173^

Evidence has shown that Cu could activate metastasis-related enzymes and signaling cascades, thus promoting the spread of cancer. For instance, the cuproenzyme LOX is involved in the invasion and metastasis of tumor cells.^174^ In estrogen receptor (ER) negative breast cancer, high expression of LOX is associated with bone metastasis and leads to the formation of tumor-driven osteolytic lesions.^175^ In tumor endothelial cells, decreased LOX expression inhibits cell migration and tube formation, and both angiogenesis and metastasis were suppressed by LOX inhibitors in an in vivo model.^176^ In an orthotopic breast cancer mouse model, knocking down *ATP7A* reduced LOX activity and reduced the recruitment of myeloid cells to the lungs, suppressing tumor growth and metastasis.^177^ The Cu chaperone ATOX1 may also be involved in the metastasis-related ATP7A-LOX pathway. A recent study using single-cell tracking analysis suggests that ATOX1 may be required for the migration of breast cancer cells, as silencing ATOX1 expression result in reduced activity of LOX and reduce the velocity and directionality of cell migration.^178^ In addition, the secreted Cu-binding glycoprotein SPARC (secreted protein acidic and cysteine-rich, also known as osteonectin) has been shown to modulate cell–matrix interactions and promote the invasion and metastasis of tumor cells.^179^ Cu also plays a role in modulating the expression of programmed death-ligand 1 (PD-L1), an immune checkpoint inhibitor associated with cancer immune evasion; specifically, depleting Cu promotes the degradation of PD-L1, thereby reducing tumor growth and improving survival in a neuroblastoma xenograft mouse model.^180^

#### Cu complexes in cancer therapy

The altered regulation of Cu homeostasis in tumors, coupled with the important role that Cu may play in promoting the progression of cancer, has led to the development of Cu-coordinating compounds for use in anticancer therapies. Two main strategies for targeting Cu homeostasis have been proposed, including the use of Cu chelators to decrease Cu bioavailability, and the use of Cu ionophores to deliver Cu into cells in order to increase intracellular Cu levels.

#### Cu chelators

A number of Cu-chelating agents such as TTM, trientine, and d-penicillamine have been developed and tested for their anti-tumor activity in both animal models as well as in clinical trials. Depletion of Cu using chelators has been shown to delay cancer metastasis by inhibiting the vascularization of lesions across several animal models, including a rabbit VX2 carcinoma model,^181^ a mouse model of hepatocellular carcinoma,^182^ and head and neck squamous cell carcinoma.^183^ The antimetastatic activity of Cu chelators may be attributed to their ability to prevent the recruitment of endothelial progenitor cells, which play an important role in angiogenesis and in the development of macroscopic metastases.^184^ In addition, depleting Cu in HER2/neu transgenic mice causes tumor shrinkage, suppresses angiogenesis, and inhibits the progression from microscopic to macroscopic tumors.^170,185^ In a mouse model of mesothelioma tumor, the use of trientine, d-penicillamine, and TTM could also exhibit inhibitory effects on tumor growth and impede tumor angiogenesis.^186^

With respect to the specificity of Cu chelators, the use of trientine has been shown to inhibit the expression of IL-8 and exhibits anti-tumor effects in hepatocellular carcinoma.^187^ Trientine also decreases CD31 expression and inhibits endothelial cell proliferation.^182^ In addition, d-penicillamine has been shown to inhibit the activity of LOX, thereby resulting in impaired collagen cross-link formation, reduced VEGF expression, and delay the progression of glioblastoma in vivo.^188^ Brem et al. have reported that combining d-penicillamine treatment with a low Cu diet reduced tumor weight and vascular density in a gliosarcoma xenograft model.^189^

Among the various Cu chelators developed to date, TTM has been well-studied in several models. For example, in RIP1-Tag2 mice, a model of pancreatic neuroendocrine tumor, administration of TTM delays the angiogenesis in premalignant lesions as well as reduces late-stage tumor growth.^159^ TTM may exert its anti-tumor effects by suppressing the transcriptional activity of NF-κB, which in turn decreases the production and secretion of downstream angiogenic factors, such as VEGF and cytokines such as IL-1α and IL-8, thereby reducing angiogenesis.^190^ In addition, TTM has been shown to induce the degradation of HIF-1α and reduce the expression of pro-angiogenic factors.^191^ TTM can also suppress Cu chaperone proteins, inhibiting the delivery of Cu to cuproenzymes such as LOX.^192^ Moreover, lowering Cu levels using TTM affects the activity of MEK1/2 kinase and BRAF-driven tumorigenesis, thus decreasing the growth of xenografted BRAF^V600E^ tumors. With respect to its clinical relevance, a phase II trial to investigate the effects of TTM in patients with malignant mesothelioma found that TTM has anti-angiogenic effects and delays disease progression in patients with stage I or stage II mesothelioma.^193^ Notably, another phase II trial found that although TTM may not improve survival in patients with kidney cancer when used as a monotherapy, it may be more effective when combined with other anti-angiogenic agents.^194^

#### Cu ionophores

Several Cu ionophores such as elesclomol, bis(thiosemicarbazone) analogs [Cu^II^(atsm) and Cu^II^(gtsm)], and disulfiram (DSF) have been shown to raise intracellular Cu levels and exert anticancer activity. The cytotoxic properties of ionophores toward cancer cells may be attributed in part to their ability to increase ROS production and/or inhibit the proteasome.^195,196^ Specifically, treatment with DSF and Cu could decrease expression of the tumor suppressor gene *PTEN* and activate AKT signaling in human breast cancers, providing a rationale for testing combination therapies using Cu ionophores and PI3K-AKT inhibitors in future studies.^197^ With respect to glioblastoma, the cytotoxic effects of the standard-of-care drug temozolomide could be augmented by the addition of a DSF–Cu complex, and the combination of DSF–Cu with temozolomide is capable of inhibiting tumor growth and improving survival in a xenograft mouse model.^198^ The DSF–Cu complex has also been shown to display cytotoxicity towards aldehyde dehydrogenase (ALDH)^+^ cancer stem cells due to its inhibitory effects on ALDH activity.^199,200^ In addition, for Cu bis(thiosemicarbazone) analogs, the anticancer activity of Cu^II^(atsm) has been demonstrated in hamsters bearing colon cancer tumors,^201^ and Cu^II^(gtsm) has been shown to exert tumor-killing effects on prostate cancer cells in vitro and significantly reduce the prostate cancer burden in an orthotopic mouse model.^202^

Elesclomol is a novel Cu ionophore that has been shown to bring Cu to the mitochondria, and subsequently, increase oxidative stress and cause cell death.^203^ As a drug candidate, elesclomol has been shown to enhance the therapeutic efficacy of paclitaxel (Taxol) in patients with refractory solid tumors and in patients with stage IV metastatic melanoma.^204^ It is noted that in the phase III clinical trial consisting of patients with advanced melanoma, the treatment of elesclomol lead to a more favorable outcome in patients with normal LDH levels compared with those patients with high LDH levels, suggesting that elesclomol may be less effective in treating cancer types with a high rate of glycolysis.^205^

### Cardiovascular disease

#### Cu in relation to atherosclerosis

Cu has also been suggested to play an important role in the pathogenesis of atherosclerosis. Epidemiological evidence has linked high serum Cu levels with an increased risk of atherosclerotic disease.^206,207^ In addition, in human atherosclerotic plaques, elevated levels of Cu have been observed.^208^ The local release of Cu ions around rat carotid arteries has been shown to promote the thickening of the neointima and the generation of arteriosclerotic lesions in response to vascular injury,^209^ whereas the use of Cu chelators exhibit inhibitory effects on vascular inflammation, the development of atherosclerotic lesions, and neointimal formation in response to vascular injury in *ApoE* knockout mice.^167,210–212^ Notably, in neointimal vascular smooth muscle cells (VSMCs) or in the intimal lesions of atherosclerotic vessels, the Cu chaperone ATOX1 and exporter ATP7A are found to be highly expressed and colocalized.^209,213–215^ Moreover, in *Atox1*^*−/−*^ mice, the expansion of extracellular matrix and neointimal formation were inhibited in response to vascular injury, accompanied by decreased accumulation of VSMCs in the neointima and reduced LOX activity.^173^

The potential mechanism by which Cu promotes atherosclerosis is currently unclear. One possibility is that Cu may play role in inflammatory responses involved in atherosclerosis. Cu deficiency reduces the expression of adhesion molecules such as ICAM-1 and VCAM-1, which mediate the adhesion of leukocytes to activated endothelial cells.^216^ In addition, Cu is capable of interacting with risk factors for the atherogenic process, such as triggering the oxidation of LDL and interacting with homocysteine to enhance hydrogen peroxidation.^217–219^ Deficiency of Cu could increase the level of total cholesterol, a key factor to increase atherosclerosis risk. Furthermore, Cu deficiency may lead to reduced NO levels by decreasing SOD1 levels, in turn promoting atherosclerosis via impaired endothelial function, reduced vasodilation, and increased oxidative stress.^220^ Given that both Cu accumulation and Cu deficiency are potentially detrimental to vascular integrity and function, maintaining Cu homeostasis is essential for preventing atherosclerosis and related cardiovascular disease.

#### Cu in relation to cardiac hypertrophy

Cardiac tissue requires high levels of Cu to maintain mitochondrial function and produce large amounts of energy needed for normal cardiac function. Indeed, it has been demonstrated in experimental animal models, the deficiency of Cu could lead to hypertrophic cardiomyopathy.^221,222^ In humans, cardiomyopathy has been observed in individuals with mutations in the synthesis of cytochrome c oxidase 2 (SCO2), a chaperone involved in trafficking Cu to cytochrome c oxidase (COX).^223^ Changes in cardiac morphology due to cardiac Cu deficiency generally include mitochondrial swelling and fragmentation, myofibril derangement, and enlarged myocytes.^224^ Functionally, impaired mitochondrial respiratory function and electrocardiographic abnormalities have also been observed in Cu-deficient hearts.^224,225^ In Cu-deficient animals and humans, cardiovascular lesions could be presented as aortic fissures and aortic rupture, possibly due to an altered architecture of elastic fibers.^226^ Cu deficiency can also alter the expression of myocardial gene profile, for example by upregulating the inflammatory cytokine TNF-α and genes involved in regulating cardiac contractility, fibrosis, and calcium cycling, all of which may underlie the changes in cardiac function under Cu-deficient conditions.^227,228^

Repletion of Cu via supplementation can reverse many of the adverse effects of Cu deficiency in the heart.^227^ In Cu-deficient mice, lipids are deposited in the myocardial tissue, with cardiac hypertrophy, mitochondrial swelling, and a blunted response to the isoproterenol, an agonist of beta-adrenergic; The repletion of Cu could reverse these changes in the heart and improve electrical conduction.^229,230^ Interestingly, in a patient with SCO2 mutations who presented with severe hypertrophic cardiomyopathy, Cu-histidine supplementation has been reported to improve heart function, showing attenuated cardiomyopathy as well as the normalization of blood pressure.^231^

Given that altered regulation of Cu, homeostasis has been associated with a variety of disease conditions, future studies are warranted in order to determine the precise mechanism by which Cu imbalance causes cellular damage, thus providing important new information that can be used to design treatments for preventing disease and/or slowing disease progression.

## Cuproptosis is distinct from other known cell death pathways

Previous studies have shown that excessive levels of trace elements can cause cell death via specific pathways. Ferroptosis, an iron-dependent form of cell death, is one such example in which excess iron triggers the accumulation of lipid peroxides in membranes, leading to programmed cell death.^232,233^ In addition to pathways involved in iron metabolism, several other molecular pathways have also been implicated in ferroptosis, including the guanosine triphosphate cyclohydrolase 1/tetrahydrobiopterin (GCH1/BH4) axis,^234^ the cysteine/glutathione/glutathione peroxidase 4 (GSH/GPX4) axis,^235,236^ and the ferroptosis suppressor protein 1/coenzyme Q (FSP1/CoQ) axis.^234,237,238^ Moreover, recent studies suggest that ferroptosis is involved in a wide range of diseases such as hemochromatosis, neurodegenerative diseases, liver fibrosis, and cardiovascular disease.^239–248^

In contrast, the mechanisms that underlie Cu-induced cell death are poorly understood. Indeed, contradictory findings suggest that excess Cu^2+^ may induce cell death via apoptosis,^249^ caspase-independent cell death, or an accumulation of ROS.^250–252^ Notably, Tsvetkov et al. recently showed that intracellular Cu induces a novel form of regulated cell death characterized by the aggregation of lipoylated mitochondrial enzymes and a loss of Fe–S proteins; they called this Cu-dependent form of cell death cuproptosis.^2^ Specifically, the authors found that treating cells with the Cu ionophore elesclomol at concentrations as low as 40 nM increased intracellular Cu levels and triggered cuproptosis.^2^ Importantly, they also showed that pharmacologically inhibiting all other known cell death pathways—including necroptosis (with necrostatin-1), ferroptosis (with ferrostatin-1), oxidative stress (with N-acetyl cysteine), and apoptosis (with Z-VAD-FMK)—failed to suppress elesclomol-induced cell death; in contrast, only Cu chelator treatment show potent rescue effect on such excess Cu^2+^-induced cell death, suggesting that cuproptosis is distinct from other forms of cell death.

### Cuproptosis is linked to altered mitochondrial enzymes

**Fig. 2:**
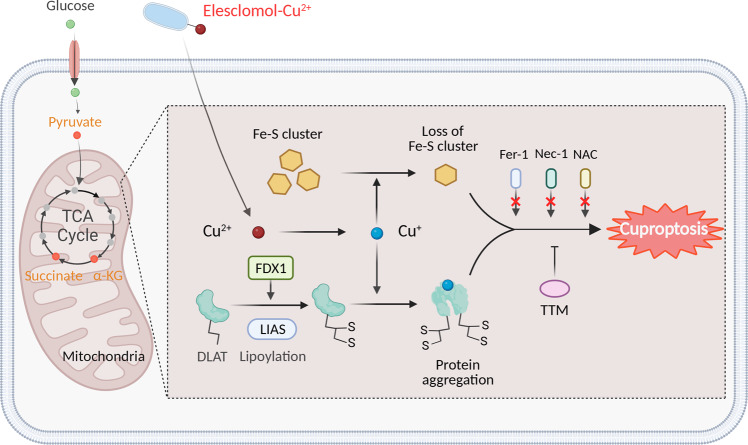
Schematic model of cuproptosis. Cu ionophores such as elesclomol bind extracellular Cu and transport it to intracellular compartments. Cu then binds to lipoylated mitochondrial enzymes in the TCA cycle such as DLAT, inducing the aggregation of these proteins. FDX1/LIAS is an upstream regulator of protein lipoylation, facilitating the aggregation of mitochondrial proteins and loss of Fe–S clusters. Together, these aberrant processes lead to proteotoxic stress and ultimately cell death. Cu chelators such as TTM inhibit cuproptosis, while inhibitors of ferroptosis (Fer-1), necroptosis (Nec-1), and oxidative stress (NAC) have no effect on cuproptosis. The solid orange circles in the TCA cycle indicate metabolites that are relevant to the lipoic acid pathway. Created with BioRender. α-KG α-ketoglutarate, DLAT dihydrolipoamide S-acetyltransferase, FDX1 ferredoxin-1, Fe–S iron–sulfur, Fer-1 ferrostatin-1, LIAS lipoic acid synthetase, NAC N-acetyl cysteine, Nec-1 necrostatin-1, TCA tricarboxylic acid, TTM tetrathiomolybdate

The mitochondrion is a major target of Cu-induced cell death, with oxidative damage to the mitochondrial membrane and impaired function of enzymes in the TCA cycle.^138,253^ In patients with Cu overload, aconitase inactivation has been attributed to reduced CCO activity and inhibition of the TCA cycle.^71^ Interestingly, metabolite profiling of cells treated with Cu ionophore presents a time-dependent increase in the dysregulation of many TCA cycle-associated metabolites, and inhibiting the electron transport chain complexes I and II significantly reduced Cu-induced cell death.^2^ Furthermore, Cu treatment selectively alters a series of metabolic enzymes via lipoylation, a highly conserved post-translational modification. Although relatively few proteins are lipoylated in mammalian cells, all lipoylated proteins are involved in the TCA cycle.^254,255^ One such lipoylated protein is dihydrolipoamide S-acetyltransferase (DLAT), a subunit of the pyruvate dehydrogenase complex.^256^ Cu can bind directly to DLAT, promoting the disulfide bond-dependent aggregation of lipoylated DLAT. Using a genome-wide CRISPR screen, mitochondrial ferredoxin (FDX1), and lipoyl synthase (LIAS) were identified as key regulators of Cu toxicity,^2,257^ and genetic knockout of either FDX1 or LIAS leads to an accumulation of pyruvate and α-ketoglutarate, reducing protein lipoylation and inhibiting Cu-induced cell death (Fig. 2).

Importantly, Cu toxicity has also been linked to a disruption of iron–sulfur (Fe–S)-containing enzymes. For example, in *Saccharomyces cerevisiae*, Cu-mediated damage to the labile Fe–S pool in mitochondrial ferredoxin leads to downstream growth inhibition.^258^ In vitro, Cu has been shown to block Fe–S cluster formation by inhibiting the activity of relevant mitochondrial assembly proteins.^259^ Moreover, the loss of the mitochondrial ABC transporter ATM1, which transports intermediates required for Fe–S cluster formation, exacerbates Cu-induced toxicity.^260^ Notably, a recent study has found that treating cells with a Cu ionophore resulted in an FDX1-dependent loss of Fe–S cluster proteins^1^ (Fig. 2). The majority of Fe–S proteins are essential cofactors for enzymes involved in the electron transport chain and other biochemical processes; thus, the aggregation of mitochondrial enzymes may disrupt the function of these Fe–S clusters and ultimately lead to cell death. Given that Cu has also been shown to destabilize Fe–S proteins in bacteria and yeast, it is reasonable to speculate that a Cu-containing complex might be developed to trigger cuproptosis in bacteria as an antimicrobial agent.^261–263^

### Cuproptosis as a putative therapeutic target for Wilson’s disease

**Fig. 3:**
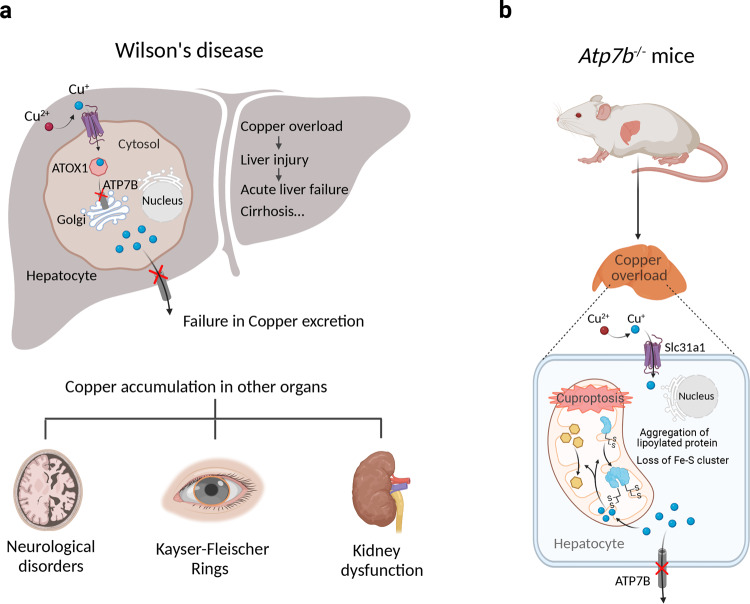
The putative role of cuproptosis in Wilson’s disease. **a** Patients with Wilson’s disease present with Cu overload in their hepatocytes. Mutations in ATP7B impair Cu loading into secretory vesicles and into cuproproteins such as ceruloplasmin. Cu excretion via the biliary tract is also impaired, resulting in an accumulation of Cu in the liver. The resulting Cu-induced toxicity leads to chronic liver disease and cirrhosis. Cu also accumulates in other tissues, including the brain, cornea, and kidneys, resulting in neurological impairment, Kayser–Fleischer rings, and impaired kidney function, respectively. **b** Atp7b knockout mice (a model of Wilson’s disease) develop Cu overload, with a loss of lipoylated and Fe–S cluster proteins in the liver, suggesting that this Cu overload has similar cellular effects as those induced by Cu ionophores, suggesting that cuproptosis may play a pathogenic role in Wilson’s disease. Created with BioRender. ATOX1 antioxidant 1 copper chaperone-1, ATP7B ATPase Cu transporter 7B, Fe–S iron–sulfur, Slc3a1 solute carrier family 3 member 1, STEAP six-transmembrane epithelial antigen of prostate

As mentioned above, mutations in the *ATP7B* gene, which encodes the Cu-transporting P-type ATPase ATP7B, are associated with Wilson’s disease, a life-threatening disorder in which patients present with progressive Cu accumulation in several tissues, particularly the liver, brain, and cornea. Importantly, Cu deposition in the liver and brain can cause cellular damage and severe hepatic and neurological symptoms.^67^ Disrupted mitochondrial membranes and increased oxidative damage have been observed in the Long–Evans Cinnamon rat, an animal model of Wilson disease.^264^ In addition, Tsvetkov et al. have shown that Atp7b knockout mice exhibit reduced levels of lipoylated enzymes and Fe–S cluster proteins compared to wild-type mice,^2^ reminiscent of the cellular phenotype induced by Cu ionophores (Fig. 3).

Cu chelation, such as trientine/d-Penicillamine, serves as an effective treatment for Wilson’s disease.^265,266^ Moreover, the Cu chelator TTM has also been evaluated in several clinical trials. For example, Brewer et al. studied 48 patients with neurological presentation and found that disease progression was reduced in the TTM-treated group compared to the trientine-treated group.^86^ In another phase II clinical trial, Weiss et al. evaluated the effect of the oral Cu–protein-binding molecule WTX101 (the bis-choline salt of TTM) and found that WTX101 rapidly improved Cu homeostasis, leading to the improved neurological outcome and stabilizing liver function^90^ (Table 1). Given that TTM inhibits the cytotoxic effects of Cu ionophores, it is reasonable to speculate that cuproptosis may play a role in the progression of Wilson’s disease. It is also worth noting that α-lipoic acid, a metabolite in the cuproptosis pathway, has shown promising results in both cell-based and animal models of Wilson’s disease.^267^ Therefore, it would be interesting to test whether novel compounds that target cuproptosis may be effective in treating this intractable disease.

**Table 1:** Overview of the clinical development of Cu-modulating agents

Agents	Action	Indication and testing stage	Ref(s)
D-penicillamine	Chelator	Wilson’s disease (approved)	^266^
Tetrathiomolybdate	Chelator	Wilson’s disease (phase II)	^86^
Trientine	Chelator	Wilson’s disease (approved)	^265^
WTX101	Chelator	Wilson’s disease (phase III)	^90^
Clioquinol	Ionophore	Fungal infection (approved)	^263^
Cu^II^(atsm)	Ionophore	Amyotrophic lateral sclerosis (phase II)	^276^
Disulfiram	Ionophore	Glioblastoma (phase I/II)	^286,287^
Elesclomol	Ionophore	Melanoma (phase III)	^206^

### Cuproptosis in relation to neurodegenerative diseases

Many studies have suggested that altered Cu homeostasis is directly related to the progression of several neurodegenerative diseases, including Alzheimer’s disease (AD), Huntington’s disease (HD), and amyotrophic lateral sclerosis (ALS).^268^ For example, high levels of Cu have been found in senile plaques and serum in patients with AD,^269,270^ and high concentrations of Cu have also been measured in Aβ plaques in the dentate gyrus subregion of the hippocampus in a mouse model of AD.^271^ On the other hand, Cu chelation treatment and knocking down the Cu transporter Ctr1 were shown to rescue neurotoxicity in a *Drosophila* model of AD.^272^ With respect to HD, an accumulation of Cu in the brain has been suggested to promote the aggregation of mutant HTT proteins, thus accelerating disease progression.^136,273^ In addition, a number of studies have suggested that restricting dietary Cu, treatment with Cu chelators, and genetic manipulation of Cu transporters may delay disease progression in animal models of HD.^141–143^ With respect to ALS, mutations in the *SOD1* gene (which encodes Cu/Zn superoxide dismutase, a physiologically important Cu-binding protein) are the principal cause of the familial form of ALS.^274,275^ Interestingly, relatively low levels of Cu have been measured in mutant SOD1 proteins, whereas increased levels of Cu have been reported in the cerebrospinal fluid of patients with ALS and mouse models of ALS.^120,125^ Notably, Roberts et al. previously reported that treating SOD1^G37R^ mice (a model of ALS) with the therapeutic Cu-delivery agent Cu^II^(atsm) restored Cu content in mutant SOD1 proteins and increased survival.^119^ The therapeutic efficacy of Cu^II^(atsm) in treating ALS has also been tested in patients^276^ (Table 1). It is also interesting to note that the Cu chelator TTM has also been shown to decrease Cu levels in the spinal cord of transgenic mice expressing the human SOD1^G93A^ mutant protein, extending their lifespan.^128^ Taken together, these findings indicate that the precise role of Cu in the progression of ALS and other neurodegenerative diseases warrants further study.

With respect to the underlying mechanism, evidence suggests that mitochondrial dysfunction may play a role in Cu-induced neurotoxicity. For example, exposing neuroblastoma cells to Cu markedly increases the production of mitochondrial ROS, followed by decreased production of pyruvate dehydrogenase in the TCA cycle and respiratory complex I.^253^ In addition, adding Cu to neuronal/glial cultures leads to a collapse in mitochondrial membrane potential (ΔΨm) and inhibits the production of α-ketoglutarate dehydrogenases and mitochondrial pyruvate, whereas the antioxidant dihydrolipoic acid attenuates these Cu-induced effects and subsequent cell death.^138^ Nevertheless, additional studies are clearly needed in order to determine whether cuproptosis plays a pathogenic role in neurodegenerative diseases discussed here and whether blocking cuproptosis may serve as a novel therapeutic strategy for treating patients with these devastating, progressive conditions.

### Potential therapeutic strategies for targeting cuproptosis in cancer

**Fig. 4:**
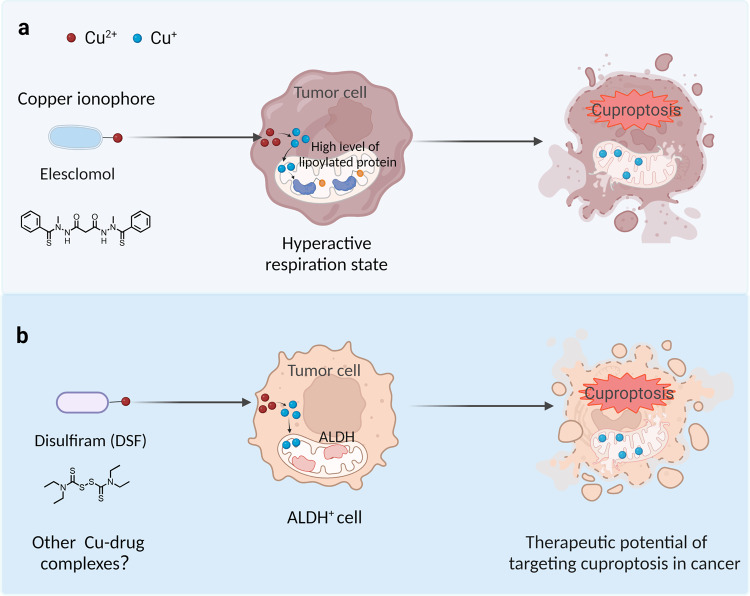
Two potential therapeutic strategies to target cuproptosis in cancer. **a** The Cu ionophore elesclomol is believed to induce cuproptosis in cancer cells that either express high levels of lipoylated mitochondrial enzymes or are in a hyperactive respiratory state. **b** Disulfiram combined with Cu selectively targets cancer cells with high ALDH expression. Created with BioRender. ALDH aldehyde dehydrogenase, DSF disulfiram

In recent decades, a growing body of evidence suggests that the Cu-complex might serve as a potential therapeutic target for treating cancer, as Cu has been shown to promote the death of cancer cells via apoptosis and/or an accumulation of free radicals.^277,278^ These findings regarding cuproptosis provide new insights into potential applications for treating cancer. Notably, Xu et al. reported the development of a glucose oxidase (GOx)-engineered nonporous Cu-coordination nanomaterial called GOx@[Cu(tz)] as a cuproptosis-based therapy in cancer.^279^ Using an in vivo system, the authors showed that GOx@[Cu(tz)] inhibited tumor growth by 92% in athymic mice with bladder tumors, with negligible systemic toxicity.^279^ In addition, Cu ionophores such as elesclomol have been shown to have anticancer activity by inducing ROS production in cancer cells.^280^ This novel finding suggests that elesclomol may have additional killing potency in cancer cells that express high levels of lipoylated mitochondrial proteins and a hyperactive respiratory state (Fig. 4). Interestingly, a randomized double-blind phase III clinical trial to evaluate elesclomol in combination with chemotherapy in patients with melanoma showed that elesclomol had stronger anti-tumor activity in patients with low plasma levels of lactate dehydrogenase^205^ (Table 1). Given that low lactate dehydrogenase levels represent a high mitochondrial metabolic state, this finding is consistent with the observation that cells with high levels of lipoylated TCA enzymes and a hyperactive mitochondrial respiratory rate are more sensitive to elesclomol. Further clinical trials are needed in order to explore the feasibility of using Cu ionophores to treat tumors with these metabolic profiles.

Disulfiram (DSF), another Cu-binding compound, has also been shown to be able to induce cuproptosis.^2^ Moreover, in vitro studies, have shown that when combined with cupric ion [Cu(II)], DSF has anti-tumor activity in a variety of cancers.^281^ Building on the previous finding that DSF inhibits aldehyde dehydrogenase (ALDH), preclinical studies have shown that the combination of DSF and Cu(II) selectively targets and kills ALDH^+^ cancer stem cells, reducing the risk of tumor recurrence.^199,282,283^ In clinical trials, the anticancer and/or chemosensitizing effects of DSF, which can cross the BBB, have been demonstrated in patients with glioblastoma.^284–286^ Moreover phase I clinical trial has shown that the combination of DSF–Cu and temozolomide has an acceptable safety profile and increases progression-free survival in patients with glioblastoma^287^ (Table 1). These findings suggest that ionophore-mediated Cu delivery to the intracellular compartment might be a promising therapeutic strategy for a subset of tumors, and additional Cu–ionophore complexes that target cuproptosis warrant further development.

## Conclusions and future perspectives

In cells, Cu acts as a double-edged sword: on one hand, Cu is an essential cofactor for many enzymes; on the other hand, excess Cu can induce oxidative stress and drive cell death. Recent studies have revealed that cuproptosis, a Cu-dependent form of cell death, is mediated by the lipoylation of mitochondrial enzymes. This novel finding provides new perspectives regarding the link between Cu-induced cell death and mitochondrial metabolism, advancing our understanding of Cu biology, and shedding new light on cell death pathways.^2^

Pioneering studies have revealed that a variety of metal ions can trigger cell death via distinct signaling pathways. For example, ferroptosis, an iron-dependent form of cell death, is characterized by excessive lipid peroxidation on cell membranes. Comparing ferroptosis and cuproptosis, it is interesting to note that mitochondria play a critical role in these two different types of cell death. Recent work has shown that mitochondrial glutathione (GSH) can slow Cu-induced cell death by suppressing enzyme lipoylation and promoting the oligomerization of DLAT. With respect to ferroptosis, the use of mitochondria-targeted ROS scavenger mitoquinone (MitoQ), which also increases GSH levels, could preserve mitochondria integrity and protect cells from lipid peroxide accumulation and subsequent cell death. On the other hand, a series of morphological changes in mitochondria, including mitochondrial shrinkage, increased membrane density, and mitochondrial fragmentation, have been observed during ferroptosis, but not in cuproptosis. In light of the finding that Cu can affect iron homeostasis and even induce ferroptosis, further study is needed in order to determine the precise morphological features of cuproptosis and to determine whether potentially relevant crosstalk exists between these two pathways.

As a newly discovered form of cell death, the precise mechanisms that underlie cuproptosis are poorly understood, although the lipoic acid pathway has been shown to play a key role in mediating cuproptosis. An interesting question is whether other metabolic pathways are also involved in cuproptosis. In addition, based on currently available data, another open question is how the aggregation of lipoylated mitochondrial enzymes triggers the Cu-dependent signaling cascades that lead to cell death. Additional studies are therefore warranted in order to identify the key players and explore the mechanisms that underlie cuproptosis, thereby providing a clear picture of cuproptosis at the cellular, tissue, and systemic levels. Identifying and characterizing the signaling pathways that regulate this newly discovered form of Cu-dependent cell death will likely offer new opportunities for clinical applications.

Given that Cu accumulation occurs in a variety of diseases and conditions, including Wilson’s disease, certain neurodegenerative diseases, and cancer, it is reasonable to speculate that cuproptosis may play a pathogenic role in these diseases and may therefore serve as a potential therapeutic target. Based on the finding that Cu chelator exhibit a potent suppression effect on Cu-induced cell death, it would be important to carry out appropriate Cu-deprivation strategies to reduce the level of intracellular Cu^2+^ ion in Cu-overload conditions, for example, using a Cu chelator, reducing dietary Cu intake, and/or genetically modifying Cu transporters. More evidence is clearly needed in order to better understand the dynamic processes by which cuproptosis initiates and facilitates disease progression.

As cuproptosis was identified only recently, no reliable biomarkers are currently available, limiting our ability to determine whether cuproptosis is involved in human pathological conditions. In this fast-growing field, the lack of cuproptosis-specific biomarkers would be a long-standing bottleneck limiting the development of cuproptosis-targeted clinical applications. Thus, reliable and sensitive biomarkers of cuproptosis are needed to be identified in different disease settings.

With respect to treatment options, both high-throughput functional screening and AI-based approaches will likely accelerate the development of new compounds that target cuproptosis. To maximize the safety and efficacy of these therapeutic compounds, pharmaceutical studies should confirm their targeted delivery to affected organs. Tackling these key hurdles will improve our understanding of the role that cuproptosis plays in various pathophysiological conditions, thus providing a clear scientific rationale for the clinical development of strategies designed to target cuproptosis in order to treat and/or prevent Cu-related diseases.
